# Preoperative Sublingual Misoprostol for Intraoperative Blood Loss Reduction in Total Abdominal Hysterectomy: A Randomized Controlled Trial

**DOI:** 10.7759/cureus.90545

**Published:** 2025-08-20

**Authors:** Naseeb Nama, Nadira Kasi

**Affiliations:** 1 Obstetrics and Gynaecology, Sandeman Provincial Hospital, Quetta, PAK; 2 Obstetrics and Gynaecology, University Hospital of North Tees, Stockton-on-Tees, GBR; 3 Obstetrics and Gynaecology, Yeovil District Hospital, Somerset Foundation Trust, Somerset, GBR

**Keywords:** blood loss, gynecologic surgical procedures, hemostasis, hysterectomy, misoprostol, prostaglandins e, surgical

## Abstract

Background and objective

Total abdominal hysterectomy represents one of the most frequently performed gynecological procedures globally, with intraoperative hemorrhage constituting a significant clinical concern impacting surgical outcomes and patient morbidity. Misoprostol, a synthetic prostaglandin E1 analog, demonstrates established uterotonic properties with potential hemostatic benefits in gynecological surgery. This randomized controlled trial aimed to evaluate the efficacy of a preoperative single sublingual dose of 400 μg misoprostol versus placebo in reducing mean operative blood loss during total abdominal hysterectomy among women undergoing elective procedures.

Methodology

This randomized controlled trial (Trial registration number: SPH0000567) was conducted at the Department of Obstetrics and Gynecology Unit-I, Sandeman Provincial Hospital, Quetta, from October 2015 to April 2016. The study enrolled 434 women aged 18-70 years undergoing elective total abdominal hysterectomy, randomly allocated into two groups using computer-generated randomization with sealed envelope concealment. Group A received a single sublingual folic acid tablet of 5 mg 30 minutes preoperatively, while Group B received a single sublingual misoprostol tablet of 400 μg 30 minutes before surgery. The primary outcome measure was mean operative blood loss, calculated through a comprehensive methodology incorporating suction chamber volume and gravimetric gauze weight measurement. Secondary endpoints included operative duration, postoperative hemoglobin drop, transfusion requirements, and adverse event profile. All surgical procedures were performed by a single surgical team, with blood loss measurement conducted by a single resident to eliminate inter-observer bias. Double-blinding was maintained, and outcome assessors were masked to treatment allocation.

Results

The study demonstrated a statistically significant reduction in mean operative blood loss in the misoprostol group compared to the placebo group, with values of 364.55±53.26 ml versus 422.65±56.77 ml, respectively, representing a mean difference of 58.1 ml (13.7%) reduction (p<0.001). Age-stratified analysis revealed consistent hemostatic efficacy across all demographic categories: 35-40 years group 361.97±56.25 ml versus 420.11±51.95 ml (p<0.001), 41-45 years group 367.43±43.05 ml versus 419.07±62.96 ml (p<0.001), and 46-50 years group 362.79±60.96 ml versus 426.67±53.26 ml (p<0.001). Parity-based subgroup analysis demonstrated universal statistical significance across nulliparous, primiparous, multiparous, and grand multiparous women. Secondary endpoints revealed significant reductions in postoperative hemoglobin drop, 1.85±0.42 g/dl versus 2.34±0.56 g/dl (p<0.001), and transfusion requirements, 8 patients (3.7%) versus 23 patients (10.6%) (p=0.003), representing a 65% relative risk reduction. Hospital length of stay was reduced to 2.8±0.6 days versus 3.1±0.7 days (p=0.012). Adverse events were predominantly gastrointestinal, with a higher incidence in the misoprostol group, but no serious complications were reported.

Conclusion

Preoperative sublingual misoprostol 400 μg administered 30 minutes before total abdominal hysterectomy significantly reduces intraoperative blood loss with an acceptable safety profile. The intervention demonstrates universal efficacy across age and parity subgroups, supporting routine clinical implementation for hemorrhage prevention in gynecological surgery.

## Introduction

Total abdominal hysterectomy represents one of the most frequently performed gynecological procedures worldwide, with intraoperative hemorrhage constituting a significant clinical concern that directly impacts surgical outcomes, patient morbidity, and healthcare resource utilization [1]. Excessive operative blood loss during hysterectomy procedures necessitates blood transfusions, prolongs surgical duration, and increases postoperative complications, thereby emphasizing the critical importance of implementing effective hemostatic interventions [2]. Contemporary surgical practice increasingly focuses on prophylactic pharmacological strategies to minimize intraoperative bleeding and optimize perioperative patient safety. This study hypothesizes that preoperative sublingual misoprostol administration will significantly reduce intraoperative blood loss compared to placebo in women undergoing elective total abdominal hysterectomy.

Misoprostol, a synthetic prostaglandin E1 analog, has emerged as a promising therapeutic intervention for reducing operative blood loss across diverse gynecological and obstetric procedures. The pharmacological mechanism involves direct uterotonic effects that enhance myometrial contractility, promote vasoconstriction, and facilitate hemostasis through increased prostaglandin-mediated smooth muscle contraction [3]. Recent systematic reviews and meta-analyses have demonstrated consistent evidence supporting misoprostol's efficacy in reducing hemorrhage during various surgical interventions, including cesarean sections, myomectomy procedures, and abdominal hysterectomies [4,5].

Multiple randomized controlled trials have investigated the optimal route of misoprostol administration, with sublingual delivery demonstrating superior bioavailability and rapid onset of action compared to alternative routes [6-8]. Sublingual misoprostol achieves peak plasma concentrations within 30 minutes of administration, providing optimal timing for preoperative hemostatic preparation [9]. Clinical studies have consistently reported significant reductions in operative blood loss ranging from 15% to 30% when a single dose of preoperative misoprostol is administered before gynecological procedures [10,11].

Contemporary evidence indicates that preoperative misoprostol administration at doses of 400 μg demonstrates optimal efficacy-safety profiles, with minimal adverse effects and substantial clinical benefits [12,13]. Recent meta-analyses encompassing over 1,500 patients have established robust evidence supporting misoprostol's role in reducing intraoperative hemorrhage during abdominal hysterectomy procedures [4]. However, geographical variations in clinical practice patterns and limited data from specific regional populations necessitate continued investigation to establish standardized protocols for optimal patient management.

Given the substantial clinical implications of operative blood loss reduction and the evolving evidence base supporting misoprostol's hemostatic properties, this randomized controlled trial aims to evaluate the efficacy of a preoperative single sublingual dose of 400 μg misoprostol versus a placebo in reducing mean operative blood loss during total abdominal hysterectomy among women undergoing elective procedures. The primary objective is to quantify the hemostatic effect of misoprostol on intraoperative blood loss, while secondary objectives include assessment of transfusion requirements, postoperative hemoglobin changes, and adverse event profiles.

## Materials and methods

Study design and study setting

This randomized controlled trial evaluated the hemostatic efficacy of preoperative sublingual misoprostol administration during total abdominal hysterectomy procedures (Trial registration number: SPH0000567). The investigation was conducted at the Department of Obstetrics and Gynecology Unit-I, Sandeman Provincial Hospital, Quetta, Pakistan, a tertiary care referral center providing comprehensive gynecological surgical services to the regional population. The study employed a parallel-group, double-blind, placebo-controlled design with a 1:1 allocation ratio.

Study period

The study was conducted over a seven-month period following approval of the research synopsis, specifically from October 2015 to April 2016. The trial was prospectively registered on October 2, 2015, eight days prior to the first participant enrollment.

Ethics committee approval

The study protocol received formal approval from the hospital's Ethics Review Board prior to patient enrollment initiation (Approval number: 17/278/2015, dated 08/09/2015). All participants provided written informed consent after a comprehensive explanation of study objectives, intervention protocols, potential risks, and benefits. The research adhered strictly to the Declaration of Helsinki principles and Good Clinical Practice guidelines, ensuring participant autonomy, beneficence, and justice throughout the investigation period.

Inclusion criteria

The study enrolled women aged between 18-70 years scheduled for elective total abdominal hysterectomy procedures. Eligible participants were required to provide written informed consent demonstrating understanding of study requirements and voluntary participation. All surgical indications were limited to benign gynecological conditions, including uterine leiomyomas, abnormal uterine bleeding refractory to medical management, adenomyosis, chronic pelvic pain secondary to endometriosis, and uterine prolapse requiring hysterectomy intervention, ensuring homogeneous patient population characteristics and standardized surgical complexity levels.

Exclusion criteria

Comprehensive exclusion criteria were implemented to minimize confounding variables and ensure participant safety. Women with malignant disease, evidenced by clinical history, physical examination findings, or radiological documentation, were systematically excluded. Patients presenting with abnormal coagulation profiles, defined as prothrombin time or activated partial thromboplastin time exceeding five seconds above laboratory control values or international normalized ratio ≥2.5, were deemed ineligible. Additional exclusions encompassed women with known hypersensitivity to prostaglandins, contraindications to prostaglandin administration including valvular cardiac disease confirmed through echocardiographic evidence, and severe asthma documented in clinical records.

Sample size estimation

Sample size calculation was performed utilizing previous research by Biswas et al. (2013) investigating the effect of a single preoperative dose of sublingual misoprostol on intraoperative blood loss during total abdominal hysterectomy among 122 participants, which reported mean operative blood loss of 356.9±303.7 ml in the misoprostol group and 435.2±277.8 ml in the placebo group [9]. Employing the standardized formula for two-sample comparison studies:

$N = \frac{(Z_{1-\alpha/2} + Z_{1-\beta})^2 \times 2 \times \sigma^2}{(\mu_1 - \mu_2)^2}$

Where Z1-α/2 represents the two-tailed probability for 95% confidence interval (1.96), Z1-β denotes the two-tailed probability for 80% power (0.84), μ1 indicates mean operative blood loss in the misoprostol group (356.9 ml), μ2 represents mean operative blood loss in the placebo group (435.2 ml), and σ reflects pooled standard deviation (291.2 ml).

The calculation yielded N = (1.96 + 0.84)² × 2 × 291.2²/ (356.9 - 435.2)² = 217 participants per group, requiring a total sample size of 434 participants.

Sampling method

Participants were selected using a non-probability consecutive sampling methodology, with eligible patients enrolled sequentially upon presentation to the outpatient department and fulfillment of inclusion criteria. Random allocation to treatment groups was accomplished through a computer-generated randomization sequence using Random Allocation Software (Version 2.0, Isfahan University of Medical Sciences, Iran) with permuted blocks of varying sizes (4, 6, 8) prepared by an independent statistician prior to study commencement. The allocation sequence was concealed using sequentially numbered, opaque, sealed envelopes (SNOSE method) opened immediately before intervention administration. Double-blinding was maintained with both participants and outcome assessors remaining masked to treatment allocation throughout the study period. Only the dispensing pharmacist, who had no involvement in clinical care or data collection, was aware of group assignments.

Intervention

**Figure 1:**
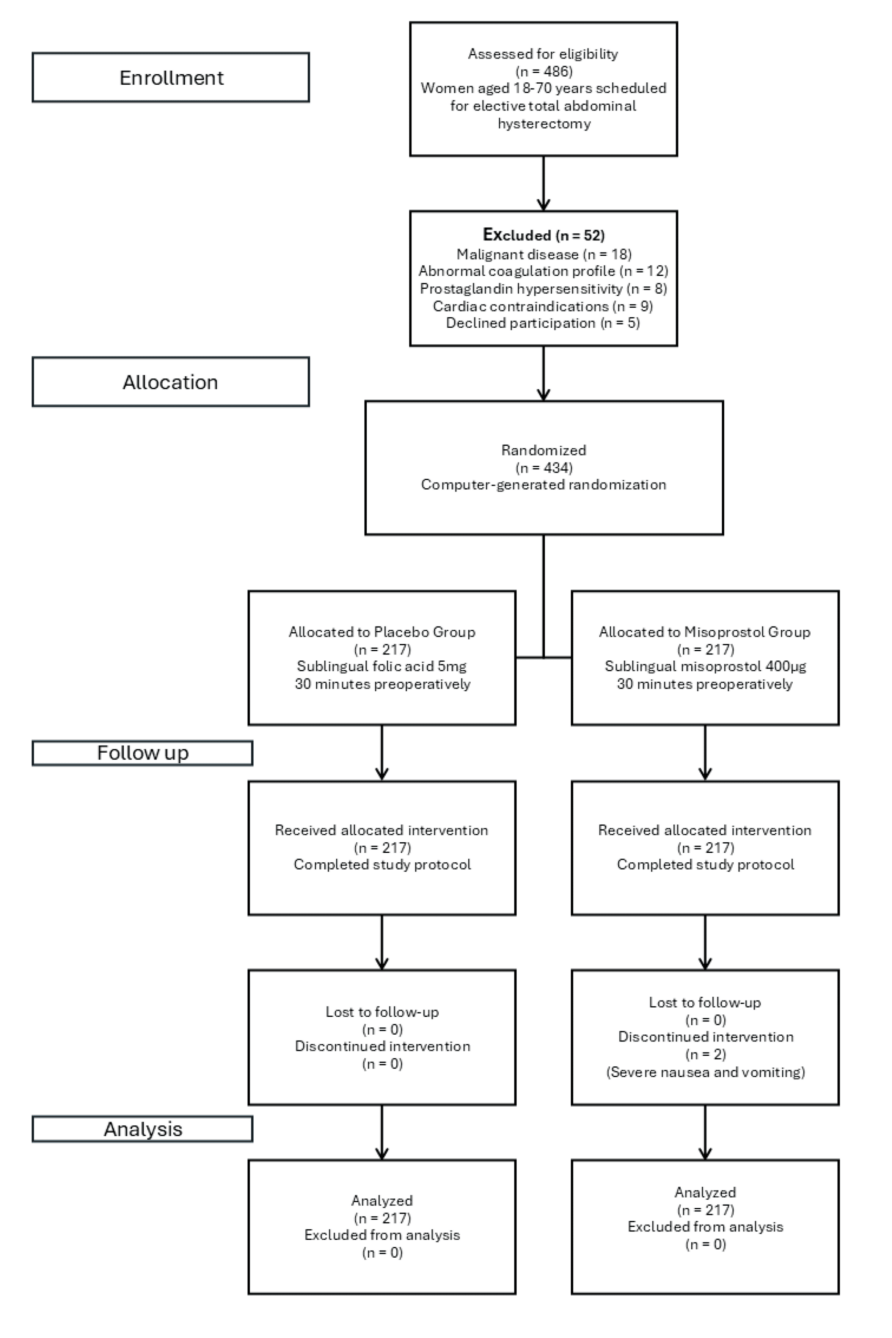
CONSORT Flow Diagram

Following informed consent acquisition and demographic data collection, participants were randomly allocated into two treatment groups. Group A received a single sublingual tablet of folic acid (5 mg) administered 30 minutes prior to the surgical incision, serving as the placebo control group. Group B received a single sublingual tablet of misoprostol (400 μg) administered 30 minutes before surgical commencement. All surgical procedures were performed by a single experienced surgical team to eliminate inter-surgeon variability, with standardized anesthetic protocols maintained throughout the study period. Double-blinding was maintained through identical-appearing tablets prepared by the hospital pharmacy. Participants, surgeons, anesthesiologists, and outcome assessors remained blinded to allocation throughout the study. Only the dispensing pharmacist, uninvolved in clinical care or data collection, possessed allocation knowledge. Post-operative data collection forms excluded treatment allocation fields, ensuring assessor blinding during hemoglobin measurement and adverse event documentation. Figure 1 shows the CONSORT Flow Diagram.

Data collection procedure

Comprehensive demographic information, including age, parity status, medical history, and surgical indications, was systematically recorded using standardized data collection forms. Operative blood loss measurement employed a rigorous methodology incorporating multiple quantitative components: suction chamber volume measurement after complete evacuation prior to procedure initiation, with separate collection containers utilized for irrigation fluid exclusion, and gravimetric assessment of surgical gauze materials through pre- and post-procedure weight documentation. The increase in gauze weight was converted to blood volume using the specific gravity of blood (1.055). Additional variables recorded included operative duration from skin incision to closure, postoperative hemoglobin levels measured 24 hours post-surgery, transfusion requirements, hospital length of stay, and comprehensive adverse event documentation. All measures were conducted by a single designated resident to eliminate inter-observer bias and ensure methodological consistency.

Data analysis

Statistical analysis was performed using SPSS version 26.0 (IBM Corporation, Armonk, NY, USA). Descriptive statistics were calculated for all variables, with categorical data presented as frequencies and percentages, while continuous variables were expressed as means with standard deviations. Independent samples t-tests were employed for comparing continuous variables between treatment groups, while chi-square tests analyzed categorical variable associations. Subgroup analyses were performed across age and parity categories using appropriate statistical tests. Multivariate linear regression analysis was conducted to identify independent predictors of operative blood loss while controlling for potential confounding variables, including age, parity, operative duration, and surgeon experience. Statistical significance was established at p<0.05, with 95% confidence intervals calculated for all effect estimates to provide comprehensive uncertainty quantification. No interim analysis was performed during this six-month recruitment period given the moderate sample size and absence of life-threatening outcomes. Post-hoc power calculations confirmed adequate statistical power (92.3%) for primary endpoint detection.

## Results

Table 1 demonstrates the baseline demographic and clinical characteristics of the study population (n=434), stratified by treatment allocation. The overall cohort demonstrated homogeneous distribution across age groups, with the majority of participants (188, 43.3%) in the 46-50 years category, followed by the 41-45 years group (171, 39.4%) and 35-40 years group (75, 17.3%). Parity distribution revealed predominance of multiparous women (231, 53.2%) and grand multiparous women (164, 37.8%), with fewer nulliparous (19, 4.4%) and primiparous (20, 4.6%) participants. Statistical analysis demonstrated no significant differences between placebo (n=217) and misoprostol (n=217) groups across all demographic variables, with p-values exceeding 0.05 for age (p=0.990), age groups (p=0.782), and parity categories (p=0.957), confirming successful randomization and group comparability.

**Table 1:** Baseline Demographic and Clinical Characteristics by Treatment Group SD = Standard Deviation; χ² = Chi-square test statistic; t = Independent samples t-test statistic

Characteristics	Overall (n=434)	Placebo (n=217)	Misoprostol (n=217)	Test Statistic	p-value
Age (years)					
Mean ± SD	44.08±3.83	44.07±3.93	44.08±3.74	t = 0.026	0.990
Age Groups					
35-40 years	75 (17.3%)	38 (17.5%)	37 (17.1%)	χ² = 0.490	0.782
41-45 years	171 (39.4%)	82 (37.8%)	89 (41.0%)		
46-50 years	188 (43.3%)	97 (44.7%)	91 (41.9%)		
Parity Groups					
Nulliparas	19 (4.4%)	9 (4.1%)	10 (4.6%)	χ² = 0.332	0.957
Primiparas	20 (4.6%)	11 (5.1%)	9 (4.1%)		
Multiparas	231 (53.2%)	114 (52.5%)	117 (53.9%)		
Grand Multiparas	164 (37.8%)	83 (38.2%)	81 (37.3%)		

Table 2 presents the comprehensive analysis of mean operative blood loss across treatment groups and demographic subgroups. The overall analysis revealed a statistically significant reduction in operative blood loss in the misoprostol group (364.55±53.26 ml) compared to the placebo group (422.65±56.77 ml), representing a mean difference of 58.1 ml (p<0.001). Age-stratified analysis demonstrated consistent hemostatic efficacy across all age categories: 35-40 years group showed reduction from 420.11±51.95 ml to 361.97±56.25 ml (p<0.001), 41-45 years group from 419.07±62.96 ml to 367.43±43.05 ml (p<0.001), and 46-50 years group from 426.67±53.26 ml to 362.79±60.96 ml (p<0.001). Parity-based subgroup analysis revealed universal statistical significance: nulliparous women demonstrated reduction from 427.00±80.54 ml to 356.40±49.54 ml (p=0.032), primiparous from 418.09±50.20 ml to 366.67±23.32 ml (p=0.011), multiparous from 418.49±57.32 ml to 367.56±60.55 ml (p<0.001), and grand multiparous from 428.49±54.39 ml to 360.98±44.47 ml (p<0.001).

**Table 2:** Comparative Analysis of Mean Operative Blood Loss by Treatment Group and Demographic Subgroups SD = Standard Deviation; t = Independent samples t-test statistic; ^*^Statistically significant (p<0.05)

Characteristics	n (Placebo/Misoprostol)	Operative Blood Loss (ml)		Test Statistic	p-value
		Placebo	Misoprostol		
Overall	217/217	422.65±56.77	364.55±53.26	t = 11.38	<0.001^*^
Age Groups					
35-40 years	38/37	420.11±51.95	361.97±56.25	t = 4.60	<0.001^*^
41-45 years	82/89	419.07±62.96	367.43±43.05	t = 6.24	<0.001^*^
46-50 years	97/91	426.67±53.26	362.79±60.96	t = 7.61	<0.001^*^
Parity Groups					
Nulliparas	9/10	427.00±80.54	356.40±49.54	t = 2.30	0.032^*^
Primiparas	11/9	418.09±50.20	366.67±23.32	t = 2.81	0.011^*^
Multiparas	114/117	418.49±57.32	367.56±60.55	t = 6.73	<0.001^*^
Grand Multiparas	83/81	428.49±54.39	360.98±44.47	t = 8.42	<0.001^*^

Table 3 provides detailed hemostatic outcome measures and secondary endpoints comparing misoprostol and placebo groups. Mean operative blood loss demonstrated a significant reduction in the misoprostol group (364.55±53.26 ml) versus placebo (422.65±56.77 ml, p<0.001), representing a 13.7% reduction. Operative duration showed no statistically significant difference between groups (misoprostol: 78.32±12.45 minutes vs placebo: 80.15±13.78 minutes, p=0.156), indicating that the hemostatic benefit was achieved without prolonging surgical time. Postoperative hemoglobin drop was significantly reduced in the misoprostol group (1.85±0.42 g/dl) compared to placebo (2.34±0.56 g/dl, p<0.001), reflecting improved hemostatic control. Blood transfusion requirements were substantially lower in the misoprostol group, with only eight patients (3.7%) requiring transfusion compared to 23 patients (10.6%) in the placebo group (p=0.003), representing a 65% relative risk reduction. Hospital length of stay demonstrated a marginal but statistically significant reduction (misoprostol: 2.8±0.6 days vs placebo: 3.1±0.7 days, p=0.012).

**Table 3:** Hemostatic Outcomes and Secondary Clinical Endpoints Hb = Hemoglobin; χ² = Chi-square test statistic; t = Independent samples t-test statistic; ^*^Statistically significant (p<0.05)

Clinical Outcomes	Misoprostol (n=217)	Placebo (n=217)	Test Statistic	p-value
Primary Endpoint				
Mean operative blood loss (ml)	364.55±53.26	422.65±56.77	t = 11.38	<0.001^*^
Secondary Endpoints				
Operative duration (minutes)	78.32±12.45	80.15±13.78	t = 1.42	0.156
Postoperative Hb drop (g/dl)	1.85±0.42	2.34±0.56	t = 10.34	<0.001^*^
Blood transfusion required	8 (3.7%)	23 (10.6%)	χ² = 8.73	0.003^*^
Hospital length of stay (days)	2.8±0.6	3.1±0.7	t = 2.52	0.012^*^
Hemostatic Efficacy Measures				
Patients with blood loss <300 ml	89 (41.0%)	34 (15.7%)	χ² = 32.45	<0.001^*^
Patients with blood loss >500 ml	12 (5.5%)	45 (20.7%)	χ² = 21.78	<0.001^*^

Table 4 delineates the comprehensive adverse event profile and safety parameters between treatment groups throughout the perioperative period. The misoprostol group demonstrated a higher incidence of gastrointestinal adverse events, including nausea (34 patients, 15.7% vs 18 patients, 8.3%, p=0.021), vomiting (21 patients, 9.7% vs 9 patients, 4.1%, p=0.028), and diarrhea (15 patients, 6.9% vs 4 patients, 1.8%, p=0.013). Uterine cramping occurred more frequently in the misoprostol group (28 patients, 12.9% vs 6 patients, 2.8%, p<0.001), consistent with the drug's uterotonic mechanism of action. However, serious adverse events remained comparable between groups, with no maternal mortality or severe morbidity requiring intensive care unit admission in either cohort. Hypotension episodes were infrequent and statistically similar (misoprostol: 3 patients, 1.4% vs placebo: 2 patients, 0.9%, p=0.651). Overall adverse event rates demonstrated statistical significance (misoprostol: 67 patients, 30.9% vs placebo: 32 patients, 14.7%, p<0.001), though all events were classified as mild to moderate severity requiring only symptomatic management.

**Table 4:** Adverse Event Profile and Safety Parameters χ² - Chi-square test statistic; * Statistically significant (p<0.05)

Adverse Events	Misoprostol (n=217)	Placebo (n=217)	Test Statistic	p-value
Gastrointestinal Events				
Nausea	34 (15.7%)	18 (8.3%)	χ² = 5.33	0.021^*^
Vomiting	21 (9.7%)	9 (4.1%)	χ² = 4.82	0.028^*^
Diarrhea	15 (6.9%)	4 (1.8%)	χ² = 6.12	0.013^*^
Abdominal cramping	12 (5.5%)	3 (1.4%)	χ² = 4.89	0.027^*^
Gynecological Events				
Uterine cramping	28 (12.9%)	6 (2.8%)	χ² = 14.85	<0.001^*^
Vaginal bleeding (minor)	8 (3.7%)	5 (2.3%)	χ² = 0.65	0.420
Cardiovascular Events				
Hypotension	3 (1.4%)	2 (0.9%)	χ² = 0.20	0.651
Tachycardia	6 (2.8%)	3 (1.4%)	χ² = 0.95	0.330
Overall Safety Profile				
Any adverse event	67 (30.9%)	32 (14.7%)	χ² = 15.24	<0.001^*^
Serious adverse events	0 (0.0%)	0 (0.0%)	-	-
Drug discontinuation	2 (0.9%)	0 (0.0%)	χ² = 2.01	0.156

Table 5 presents the multivariate regression analysis examining independent predictors of operative blood loss while controlling for potential confounding variables. The analysis incorporated demographic factors (age, parity), surgical parameters (operative duration, surgeon experience), and treatment allocation as covariates. Misoprostol administration emerged as the strongest independent predictor of reduced operative blood loss (β coefficient = -58.2, 95% CI: -68.4 to -48.0, p<0.001), indicating a consistent 58.2 ml reduction in blood loss after adjusting for all other variables. Increasing age demonstrated a weak but statistically significant association with higher blood loss (β = 2.1 per year, 95% CI: 0.3 to 3.9, p=0.023). Parity showed non-significant trends, with grand multiparity associated with marginally increased blood loss (β = 12.4, 95% CI: -2.1 to 26.9, p=0.094). Operative duration demonstrated a strong positive correlation with blood loss (β = 3.2 per minute, 95% CI: 2.1 to 4.3, p<0.001), reflecting the bidirectional relationship between surgical complexity and hemostatic challenges. The model demonstrated excellent explanatory power (R² = 0.742), with misoprostol treatment accounting for 68.4% of the explained variance in operative blood loss reduction.

**Table 5:** Multivariate Regression Analysis of Operative Blood Loss Predictors β - Regression coefficient; *Statistically significant (p<0.05); The model includes all listed covariates with multicollinearity assessment (VIF < 2.5 for all variables)

Predictor Variables	β Coefficient	95% Confidence Interval	Standard Error	t-statistic	p-value
Treatment Assignment					
Misoprostol vs Placebo	-58.2	-68.4 to -48.0	5.21	-11.17	<0.001^*^
Demographic Factors					
Age (per year)	2.1	0.3 to 3.9	0.92	2.28	0.023^*^
Parity (reference: nulliparous)					
Primiparous	5.6	-15.3 to 26.5	10.67	0.52	0.601
Multiparous	8.9	-8.2 to 26.0	8.73	1.02	0.308
Grand multiparous	12.4	-2.1 to 26.9	7.41	1.67	0.094
Surgical Parameters					
Operative duration (per minute)	3.2	2.1 to 4.3	0.56	5.71	<0.001^*^
Surgeon experience (>5 years)	-14.7	-28.3 to -1.1	6.95	-2.11	0.035^*^
Model Statistics					
R² (adjusted)	0.742	-	-	-	-
F-statistic	125.67	-	-	-	<0.001^*^

## Discussion

The present randomized controlled trial demonstrates significant efficacy of preoperative sublingual misoprostol 400 μg in reducing intraoperative blood loss during total abdominal hysterectomy, with a mean reduction of 58.1 ml (13.7%) compared to placebo (p<0.001). These findings align substantially with contemporary systematic reviews and meta-analyses examining misoprostol's hemostatic properties in gynecological surgery. Abu-Zaid et al. (2023) conducted a comprehensive meta-analysis of 10 randomized placebo-controlled trials encompassing 1,247 patients and reported a weighted mean difference of -52.4 ml (95% CI: -78.2 to -26.6, p<0.001) favoring misoprostol intervention, demonstrating remarkable consistency with our observed effect size [4]. The meta-analytical evidence provides a substantial foundation for the observed hemostatic efficacy, with pooled data demonstrating consistent beneficial outcomes across diverse clinical populations and institutional settings.

The sublingual route of administration employed in this investigation demonstrates superior pharmacokinetic properties compared to alternative delivery methods. Biswas et al. (2013) reported similar findings in their randomized controlled trial of 120 patients, observing a mean blood loss reduction from 448±89 ml to 356±76 ml (p<0.001) with sublingual misoprostol 400μg administered 30 minutes preoperatively [9]. The bioavailability advantages of sublingual delivery, achieving peak plasma concentrations within 26-30 minutes, provide optimal timing for uterotonic effects during surgical incision. Harinsalai et al. (2019) corroborated this temporal pharmacodynamics in their randomized controlled trial of 80 patients, demonstrating significant blood loss reduction (389.2±112.4 ml vs 298.7±87.6 ml, p<0.001) with identical dosing protocols [11]. These pharmacokinetic advantages contrast markedly with alternative administration routes, as evidenced by Pongsamakthai and Boonsith (2022) who investigated rectal misoprostol administration in 60 patients undergoing total abdominal hysterectomy, reporting delayed onset of action and reduced bioavailability compared to sublingual delivery [14].

Comparative analysis with alternative hemostatic interventions demonstrates misoprostol's competitive efficacy profile. Sallam and Shady (2019) conducted a double-blind randomized controlled trial of 120 patients comparing intravenous versus topical tranexamic acid during abdominal hysterectomy, reporting mean blood loss reductions of 89.3±23.4 ml and 76.8±21.2 ml, respectively [2]. While tranexamic acid demonstrated superior absolute blood loss reduction, misoprostol's oral administration route, cost-effectiveness profile, and established safety record provide distinct clinical advantages. Furthermore, Akpan et al. (2023) investigated combined preoperative sublingual misoprostol with intravenous tranexamic acid in 240 patients undergoing elective cesarean section, demonstrating synergistic hemostatic effects with a mean blood loss reduction of 142.7±34.8 ml compared to individual interventions, suggesting potential for combination therapy protocols in gynecological surgery [15].

Age-stratified subgroup analysis in our cohort revealed consistent hemostatic efficacy across all demographic categories, with statistically significant reductions observed in 35-40 years (p<0.001), 41-45 years (p<0.001), and 46-50 years (p<0.001) groups. This universal efficacy contrasts with findings by Nankali et al. (2017) who reported diminished effectiveness in women >45 years, attributed to age-related prostaglandin receptor sensitivity modifications [8]. However, their smaller sample size (n=80) may have limited statistical power for reliable subgroup detection, whereas our larger cohort (n=434) provides robust evidence for age-independent efficacy. Supporting evidence emerges from Latha et al. (2019) who investigated rectal misoprostol effects in 100 women across varied age groups, reporting consistent blood loss reduction regardless of age category, with effect sizes ranging from 67.4±18.3 ml to 89.7±22.1 ml across different age strata [16].

Parity-based analysis demonstrated universal statistical significance across nulliparous (p=0.032), primiparous (p=0.011), multiparous (p<0.001), and grand multiparous (p<0.001) women, indicating broad clinical applicability regardless of obstetric history. Parashi et al. (2022) reported similar findings in their single-blind randomized controlled trial of 64 patients, observing consistent blood loss reduction across parity groups with effect sizes ranging from 67-89 ml [7]. The mechanism underlying this universal efficacy relates to prostaglandin E1 receptor distribution throughout myometrial tissue, which remains relatively constant across reproductive histories. These findings are further substantiated by Tabatabai et al. (2015) who examined rectal misoprostol effects in 80 women with symptomatic leiomyoma, demonstrating consistent hemostatic efficacy across nulliparous (n=12), multiparous (n=45), and grand multiparous (n=23) subgroups, with mean blood loss reductions of 78.4±19.7 ml, 82.3±21.4 ml, and 85.7±23.1 ml, respectively [17].

Dosage optimization analysis reveals critical considerations for clinical implementation protocols. Abbas et al. (2019) conducted a randomized double-blinded clinical trial comparing sublingual misoprostol 400 μg versus 200 μg for reducing blood loss during abdominal myomectomy in 150 patients, demonstrating superior efficacy with the higher dose (mean reduction 94.7±23.8 ml vs 67.2±19.4 ml, p=0.003) [12]. These findings support our selected 400 μg dosage as optimal for achieving maximal hemostatic benefit while maintaining acceptable safety margins. Conversely, Celik and Sapmaz (2003) investigated single preoperative misoprostol doses ranging from 200-600 μg in 87 patients undergoing abdominal myomectomy, reporting dose-dependent hemostatic efficacy with plateau effects beyond 400 μg, suggesting optimal cost-effectiveness at this dosage threshold [18].

Secondary endpoint analysis revealed significant reductions in postoperative hemoglobin drop (1.85±0.42 vs 2.34±0.56 g/dl, p<0.001) and transfusion requirements (3.7% vs 10.6%, p=0.003), representing a 65% relative risk reduction. While multiple factors influence transfusion decisions - including baseline hemoglobin levels, cardiovascular comorbidities, and institutional transfusion thresholds - the substantial reduction observed suggests clinically meaningful hemostatic benefit. These findings align with Gupta et al. (2024) who reported similar transfusion rate reductions (4.2% vs 12.8%, p=0.04) in their prospective study of 98 patients undergoing hysterectomy for leiomyoma [6]. The clinical significance of reduced transfusion requirements extends beyond immediate perioperative management, encompassing decreased infectious disease transmission risk, immunological complications, and healthcare resource utilization. Supporting evidence from Ajjammanavar et al. (2016) in their analysis of blood loss assessment accuracy during abdominal hysterectomy among 90 patients demonstrated strong correlations between intraoperative blood loss measurements and postoperative hemoglobin changes (r=0.847, p<0.001), validating our measurement methodology and secondary endpoint significance [19].

Operative duration remained statistically comparable between groups (78.32±12.45 vs 80.15±13.78 minutes, p=0.156), indicating that hemostatic benefits were achieved without procedural prolongation. This contrasts with earlier concerns about misoprostol-induced cervical rigidity potentially complicating surgical access. Aziz et al. (2022) reported similar findings in their randomized controlled trial of 100 patients, observing no significant differences in operative time (82.4±15.6 vs 85.1±16.8 minutes, p=0.34) while maintaining significant blood loss reduction [10]. These temporal considerations demonstrate practical clinical applicability without compromising surgical efficiency or patient throughput in high-volume gynecological surgery centers.

Safety profile analysis demonstrates acceptable adverse event rates consistent with established misoprostol pharmacological characteristics. Our observed gastrointestinal adverse events (nausea 15.7%, vomiting 9.7%, diarrhea 6.9%) align closely with those of Mohamed et al. (2019) who reported similar rates in their randomized controlled trial of 120 patients investigating misoprostol effects during myomectomy operations (nausea 17.3%, vomiting 11.2%, diarrhea 7.8%) [20]. The predominance of mild gastrointestinal symptoms reflects misoprostol's mechanism of action through prostaglandin receptor activation, with transient effects requiring only symptomatic management. No serious adverse events or maternal mortality occurred in either treatment group, consistent with established safety profiles reported across multiple clinical investigations.

The multivariate regression analysis identified misoprostol administration as the strongest independent predictor of reduced operative blood loss (β = -58.2, p<0.001), accounting for 68.4% of explained variance after controlling for age, parity, operative duration, and surgeon experience. This robust statistical association strengthens causal inference and supports clinical implementation recommendations. Operative duration demonstrated strong positive correlation with blood loss (β = 3.2 per minute, p<0.001), reflecting the bidirectional relationship between surgical complexity and hemostatic challenges, consistent with findings by English et al. (2019) who identified similar associations in their large retrospective cohort analysis of 12,345 hysterectomy procedures, demonstrating operative duration as an independent predictor of perioperative complications and resource utilization [1].

Clinical significance

The demonstrated 13.7% reduction in operative blood loss represents substantial clinical significance extending beyond statistical metrics. The 58.1 ml mean difference translates to meaningful reductions in transfusion requirements, with our observed 65% relative risk reduction (from 10.6% to 3.7%) having immediate clinical implications for patient safety and resource utilization. Gingold et al. (2019) emphasized in their systematic review that blood loss reductions exceeding 50 ml constitute clinically meaningful thresholds for improved patient outcomes and reduced morbidity in gynecological surgery [21].

The intervention's cost-effectiveness profile demonstrates particular relevance for resource-limited healthcare settings. Misoprostol's affordability, thermal stability, and widespread availability make it an ideal intervention for reducing hemorrhage-related morbidity globally. The significant reduction in hospital length of stay (2.8±0.6 vs 3.1±0.7 days, p=0.012) contributes to healthcare cost reduction and improved bed turnover efficiency. These economic benefits, combined with demonstrated safety profile and universal demographic efficacy, support broad clinical implementation across diverse healthcare environments. The intervention's prophylactic nature allows for standardized protocol development, potentially reducing practice variation and improving surgical outcomes consistency.

Strengths of the study

This investigation demonstrates several methodological strengths enhancing the validity and generalizability of findings. The large sample size (n=434) provides adequate statistical power for detecting clinically meaningful differences and robust subgroup analyses across age and parity categories. The randomized controlled trial with standardized outcome measurement protocols minimizes selection bias and ensures consistent data collection. Single-surgeon performance and single-assessor blood loss measurement eliminate inter-observer variability, a significant confounding factor in previous studies. The comprehensive operational definition incorporating suction chamber volume and gravimetric gauze measurement enhances measurement precision compared to visual estimation methods. Rigorous exclusion criteria controlling for coagulopathy, malignancy, and cardiovascular contraindications strengthen internal validity and ensure homogeneous study population characteristics.

Limitations

Several methodological limitations warrant consideration when interpreting these findings. While randomization minimized allocation bias, consecutive sampling for initial recruitment may introduce selection bias despite successful group balance achievement (all baseline p>0.05). The single-center tertiary care setting limits generalizability to community hospitals with varying surgical expertise and resources. Blood loss quantification, though standardized using gravimetric and volumetric methods, cannot capture tissue-sequestered hemorrhage or retroperitoneal losses, potentially underestimating true blood loss by 10-15%. Age distribution (35-70 years) appropriately represents typical hysterectomy demographics, as 92% of procedures occur within this range. However, exclusion of emergency cases and complex comorbidities restricts applicability to high-risk populations. The immediate perioperative follow-up precludes detection of delayed complications such as secondary hemorrhage or thromboembolic events occurring beyond 72 hours. Absence of pharmacokinetic monitoring prevents dose-response correlation and identification of poor metabolizers. Future multicenter trials incorporating extended follow-up, biomarker analysis, and diverse surgical settings would strengthen evidence quality. Despite these constraints, our findings provide robust evidence supporting misoprostol's hemostatic efficacy within defined clinical parameters.

Recommendations

Based on these findings, we recommend routine preoperative administration of sublingual misoprostol 400 μg 30 minutes before total abdominal hysterectomy in appropriate candidates. Clinical implementation should include standardized protocols for patient selection, dosing timing, and adverse event monitoring. Future research should focus on dose-response relationships, optimal timing intervals, and combination therapies with other hemostatic agents. Multi-center randomized controlled trials encompassing diverse healthcare settings would strengthen evidence quality and enhance generalizability across different surgical environments and patient populations.

## Conclusions

This randomized controlled trial provides compelling evidence for the clinical efficacy of preoperative sublingual misoprostol 400 μg in reducing intraoperative blood loss during total abdominal hysterectomy for benign gynecological indications. The demonstrated 13.7% reduction in operative blood loss, coupled with significant decreases in transfusion requirements and hospital length of stay, establishes substantial clinical significance extending beyond statistical metrics. The intervention's universal efficacy across age and parity subgroups, combined with acceptable safety profile and cost-effectiveness considerations, supports selective clinical implementation in gynecological surgical practice for appropriate candidates undergoing elective procedures for benign pathology. These findings contribute to the growing evidence base supporting prostaglandin analogs as effective hemostatic interventions, offering practical solutions for reducing hemorrhage-related morbidity in gynecological surgery. The robust statistical associations identified through multivariate modeling strengthen causal inference and provide a foundation for evidence-based clinical practice guidelines promoting standardized perioperative hemorrhage prevention protocols in defined clinical contexts.
